# Throwing Injury Prevention Strategies with a Whole Kinetic Chain-Focused Approach

**DOI:** 10.1007/s12178-022-09744-9

**Published:** 2022-04-07

**Authors:** Michael Mayes, Madeleine Salesky, Drew A. Lansdown

**Affiliations:** Department of Orthopedic Surgery, Sports Medicine & Shoulder Surgery, University of California, San Francisco, 1500 Owens Street, San Francisco, CA 94158 USA

**Keywords:** Baseball, Throwing motion, Pitching mechanics, Kinetic chain, Shoulder, Injury prevention, Warm-up

## Abstract

**Purpose of Review:**

This review examines the relationship between the baseball pitching motion and the kinetic chain. The goal was to determine the underlying causes of a deficiency in throwing mechanics throughout a throwing motion, and to provide an evidence-based approach on how to prevent injuries caused by a lack of proper mechanics. In doing so, we sought to provide a warm-up strategy that can be added to every baseball player’s daily on-field routine that is tailored to each phase of the throwing motion.

**Recent Findings:**

To help minimize the risk of injury to overhead throwing athletes, a thorough understanding of the throwing motion is critical. Throwing a ball places extreme stress on the body, notably the shoulder and elbow joints. With a clear understanding of the biomechanics of throwing, we can develop an injury prevention routine to minimize unnecessary stresses throughout the kinetic chain.

**Summary:**

The throwing cycle is a complex motion that places various stresses throughout the thrower’s body, from the ankle to the core, and from the back to the shoulder and elbow. A thorough understanding of the mechanics of this motion, along with specific exercises to target the specific actions of each phase, may allow for throwers, regardless of their age and experience, to minimize injury risk.

## Introduction

Baseball and softball are among the most common sports for children and adolescents, with over 5 million children participating annually in the USA [1]. Throwing a ball places extreme stress on the shoulder and elbow joints [2–5]. The throwing motion, however, also requires careful coordination and strength throughout the entire kinetic chain [6]. With baseball increasingly becoming a year-round sport, overuse injuries are becoming more common, and youth pitchers who throw more than 100 innings per calendar year have a 3.5 times greater risk of sustaining a serious sports-related injury [7]. While USA Baseball has published pitching safety guidelines and regulations, sports-related injury among overhead throwing athletes is still common. Hamstring, lumbar paraspinal, and oblique muscle strains represent three of the five most common injuries in a report on Major and Minor League Baseball Players with data from the Major League Baseball (MLB) Health and Injury Tracking System (HITS) [8]. This potential for injury throughout the kinetic chain emphasizes the importance of a comprehensive approach to injury prevention in addition to pitch count restrictions to allow for players to continue to safely participate in throwing sports. The majority of shoulder injuries in sports are caused by repetitive overhead motion leading to overuse injuries [9, 10]. Injuries in the throwing athlete often lead to missed time for treatment and decreased performance capabilities, highlighting the need for effective injury prevention programs [11, 12].

A clear understanding of the biomechanics of throwing is essential for sports medicine specialists, including athletic trainers, physical therapists, and physicians, to effectively care for throwing athletes. The six phases of throwing include windup, stride, arm cocking, acceleration, deceleration, and follow-through [3, 13]. Deficiencies at any point in this cycle may transfer increased stress to the thrower’s shoulder and elbow or may stem from problems with total shoulder rotation, external rotation weakness, and scapular dyskinesis. Understanding the dynamic phases of throwing is imperative to understanding the development of overuse injuries in the overhead athlete [14•]. Each phase localizes force to different parts of the body, leading to potential injury in that phase. Given these differential stresses in each phase, there is also an opportunity to target specific exercises for the muscle groups active during a given phase of the throwing cycle.

The purpose of this article is to provide an evidence-based review of injury prevention strategies based on each phase of the throwing cycle that may be incorporated by healthy throwing athletes. The objectives of this paper are to describe the complex motion and muscular activation in each phase of throwing, to demonstrate how variability in throwing mechanics can contribute to sports-related injury, and to identify exercises to target each phase of throwing to limit injury risk.

## Windup

The windup phase begins with the pitcher’s first movement from the static position of facing the batter with both feet on the mound and is completed when the lead leg reaches maximum knee height [15]. Muscular activations will be seen in the iliopsoas, rectus femoris, pectineus, and sartorius to elevate the stride leg [13]. The final moment during the windup when the knee is at maximum height is referred to as the “balance point” [15]. Subsequently, the pitcher starts to remove the ball from the glove in order to begin the next phase.

The risk of injury during this time is relatively low, but the pitcher is setting the timing and tone for the remainder of the pitching motion [15]. While in this “balance point” position, we should see the pitcher’s shoulders aligned between home plate and second base demonstrating a stable center of gravity (COG) [15]. When observing the throwing motion, special attention should be focused on stability at the balance point, as alterations at this early point may result in unnecessarily increased stress through the upper extremity. The primary contributors to successfully completing the windup phase arise from strength and proprioception in the lower extremity.

**Figure 1:**
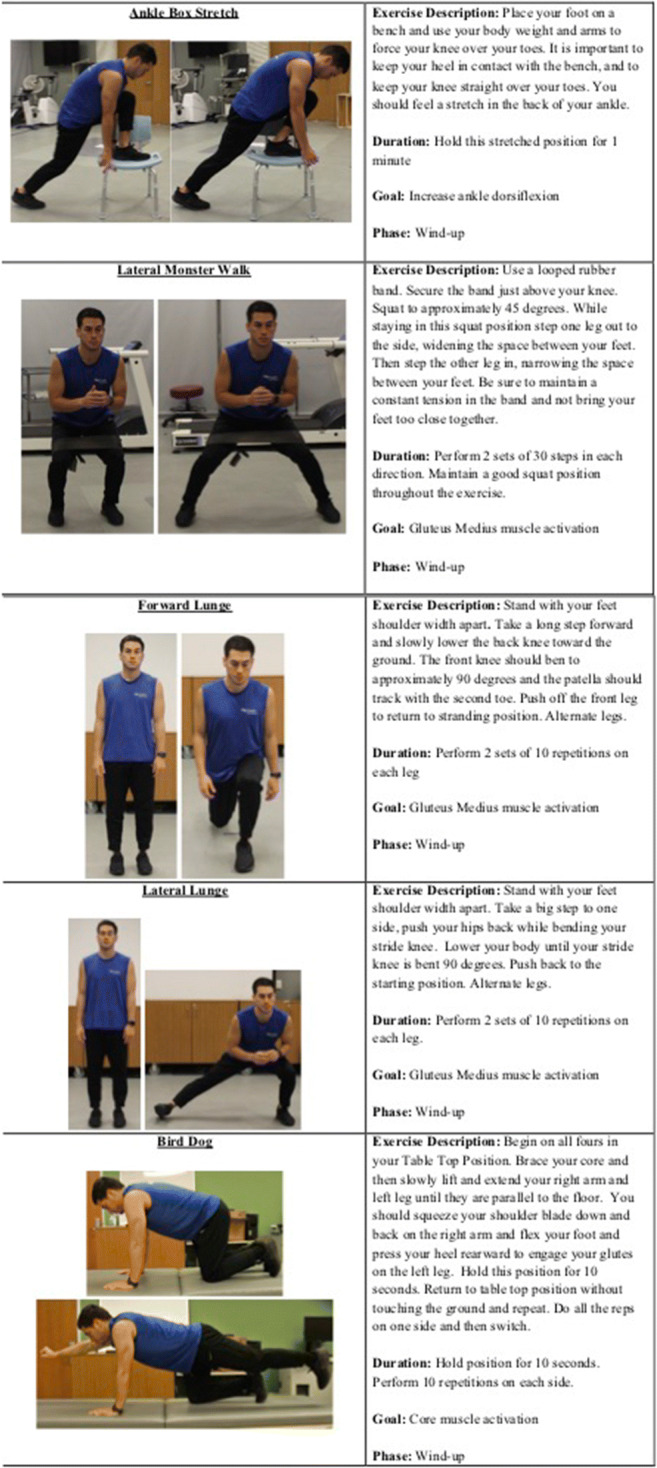
Specific stretches and exercises to help assist with flexibility, hip strength, and core strength

First, the ankle joint contributes to forming a stable foundation for the stance leg. Athletes with chronic ankle instability demonstrate increased postural sway, which can result in loss of control during the windup phase [16–19]. The primary mechanical impairments from an unstable ankle are increased anterior joint laxity [20], reduced posterior talar glide [21], and reduced range of motion, most notably in decreased dorsiflexion range of motion [22–24]. Restrictions in the anterior-posterior glide of the talus on the tibia have been well documented in those with lateral ankle instability [25]. These restrictions can be seen in those who have only experienced one incidence of lateral ankle sprain, or those who suffer from chronic ankle instability [25]. Arthokinematic restrictions may not allow the thrower to achieve the necessary dorsiflexion range of motion, which in turn can affect their COG within the windup phase. Dorsiflexion range of motion has been shown to have a significant influence of dynamic balance regardless of a history of lateral ankle sprains [26]. An ankle box stretch (Figure 1) should be a part of every pitcher’s warm-up routine to maintain necessary flexibility.

Additionally, the tensor fascia latae, gluteus medius, and gluteus minimus must isometrically contract to maintain a stable pelvis [13]. If the COG is positioned too far posteriorly or anteriorly, torque will transfer to the upper extremity, thus predisposing the shoulder and elbow to injury [15]. Factors that can contribute to faulty windup mechanics include poor balance at maximum knee height secondary to reduced lower extremity strength, poor trunk control, and tilting of the COG [15].

As the hips develop power throughout the windup phase, it is transferred through the lumbopelvic region and finally to the throwing arm [27]. The lumbopelvic muscles show significant activity during the pitching motion [28, 29]. Poor lumbopelvic control has been associated with increased shoulder horizontal abduction torque and elbow valgus torque, both of which may result in increased injury risk and decreased performance [30•]. Additionally, there is increased compressive force in the glenohumeral joint with increased pelvic tilt toward the throwing side, increased pelvic axial rotation velocity, and a decreased stride length [31]. From an athlete performance standpoint, Chaudhari et al. [32] found that pitchers with less lumbopelvic control produced more walks and hits per inning when compared to those with more lumbopelvic control. Likewise, pitchers with decreased lumbopelvic control have been shown to have an increased likelihood of injury and spending more time on the disabled list [33].

Hip and core strength are directly linked to dynamic balance [34–39]. Evaluation of stability can be done through tests like the Star Excursion Balance Test (SEBT) and the Y Balance test [40]. Deficiencies in balance are treatable through short courses of training programs. For instance, prior studies have demonstrated significant improvement in SEBT with a range of 5- to 12-week core training protocols [39, 41, 42]. Exercises like banded side-walks (“lateral monster walks”) for gluteus medius strength and activation [43] and bird dogs [44] for core stability and strength are easy to incorporate into on-field practice routines without excessive equipment. Forward lunges and lateral lunges are also excellent exercises to activate and strengthen the gluteus medius [43] (Figure 1).

## Stride

The stride phase begins with the throwing shoulder horizontally abducting to approximately 90° and ends with the front foot striking the ground [13]. From the ground up, the athlete will be engaging their ankles, legs, pelvis, core, thoracic spine, and shoulder [15, 45, 46]. Again, especially with sufficient injury history, a clinician may begin to see breakdowns within the throwing motion and kinetic chain already. Areas of common concern at this point include core strength, lumbar extension, and thoracic spine rotation. Deficiencies in the kinetic chain in these early portions of a throwing motion can change mechanics later on through the “catch-up phenomenon” and result in an altered delivery and possible injury [15, 45, 46]. Low back pain (LBP) and/or injury may develop as a consequence to poor core muscle activation/stability, which in turn may cause deficiencies later within the throwing motion [47].

LBP is a significant issue for baseball players, as it can lead to missed participation time and early career termination [48]. The prevalence of LBP in active baseball players ranges between 3 and 15% [49–53]. This pain can present itself in forms of stress reactions, stress fractures, vertebral disc degeneration, and mechanical LBP involving surrounding musculature. Each is products of repetitive, high-velocity spinal movement, and loading. For instance, Hangai et al. found that 59.7% of the tested population of Japanese baseball players had radiographic disc degeneration at one or more levels [54]. Improper mechanics may cause forces to concentrate in these regions and subsequently place throwers at risk for injury. Toyoshima et al. reported that the trunk contributes to as much as 50% of the kinetic energy and force production during the entire throwing motion [55]. Wasser et al. [56] discussed the importance of maintaining a neutral spine by avoiding excessive lumbar extension and rotation during the stride and early cocking phases.

To maintain a neutral spine through the throwing cycle, throwers must maintain lumbar stability and thoracic mobility. Additionally, the risk of LBP may be limited by combining core stability with thoracic mobility exercises better than using core exercises alone [57–60]. This approach effectively reduces the stress caused by excessive movement of segments from lumbar instability by mobilizing segments above, as found by Yang et al., Kaltenborn et al., and Sung YB et al. [57, 59, 60]. During the stride and cocking phase, instead of motion and force coming from the lumbar region, causing excessive lumbar extension and rotation, thoracic flexibility may allow for force distribution and decrease stress concentration.

**Figure 2:**
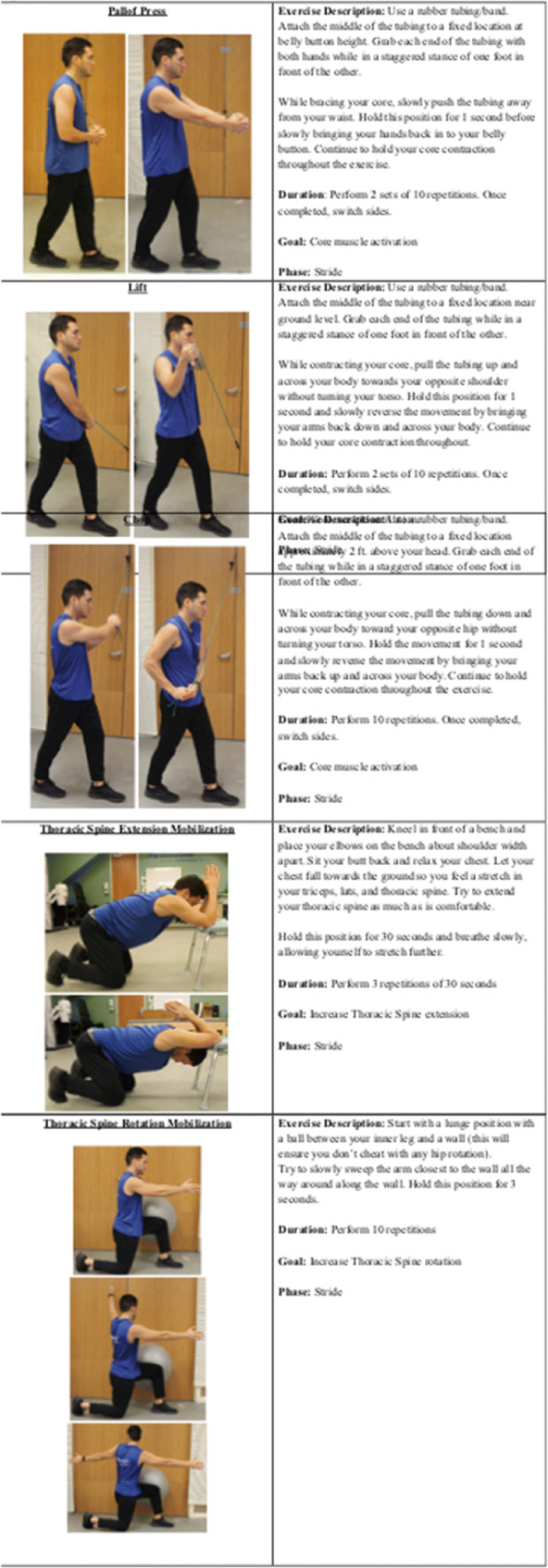
Specific exercises that allow for proper lumbar spine stability and thoracic spine mobility

Specific exercises that allow for proper lumbar spine stability and thoracic spine mobility include bird dogs [44], pallof presses, lifts, and chops, in addition to thoracic spine rotation mobilization, and thoracic spine extension mobilization (Figure 2).

## Cocking

The cocking phase begins with the front foot striking the ground and ends with maximum shoulder external rotation at 150 to 180° [61, 62]. This stage of throwing can be further divided into early and late cocking phases. Potential energy is accumulated in the early cocking phase and transferred to the throwing arm in the late cocking phase to prepare for acceleration and ball release [63].

## Early Cocking

The early cocking phase begins with lead foot contact. The quadriceps of the lead leg contracts to stabilize a fulcrum point [61, 64]. The pelvis then rotates toward home plate. Trunk rotation and extension lag behind pelvic rotation, transferring energy from the pelvis to the upper torso [3, 65]. During rotation, the abdominal and oblique musculature is activated to stabilize the trunk through the delay between pelvic and upper torso rotation [61].

Significant shoulder muscle activity is required to stabilize the throwing arm as the trunk rotates and extends. During this phase, the deltoid muscle activates to maintain 90° abduction of the throwing arm with the elbow flexed at 90 to 100° [62, 64]. The rotator cuff muscles achieve high activity to resist the compressive force generated by the trunk [62]. The shoulder girdle muscles (levator scapulae, serratus anterior, trapezius, rhomboids, and pectoralis minor) are activated to stabilize the scapula and glenoid for subsequent humeral head external rotation [61]. Importantly, the scapula must protract and rotate upwards to ensure that the humeral head is positioned in the “safe zone” on the glenoid as rotation of the throwing arm lags behind the torso [66].

Scapular dyskinesis describes a disruption of normal scapular kinematics and is a major cause of injury during the early cocking phase. At rest, excessive scapulothoracic protraction and upward rotation lead to misalignment between the scapula and glenoid [66]. While throwing, abnormal scapular movement results in a loss of coordination between the glenohumeral and scapulothoracic joints. Scapular dyskinesis has been associated with shoulder pain, shoulder impingement syndrome, rotator cuff tendinopathy, and rotational deficits which disturb the scapulohumeral rhythm [67, 68]. A meta-analysis found that asymptomatic athletes with shoulder dyskinesis had a 43% higher chance of developing shoulder pain than athletes without scapular abnormalities [69].

**Figure 3:**
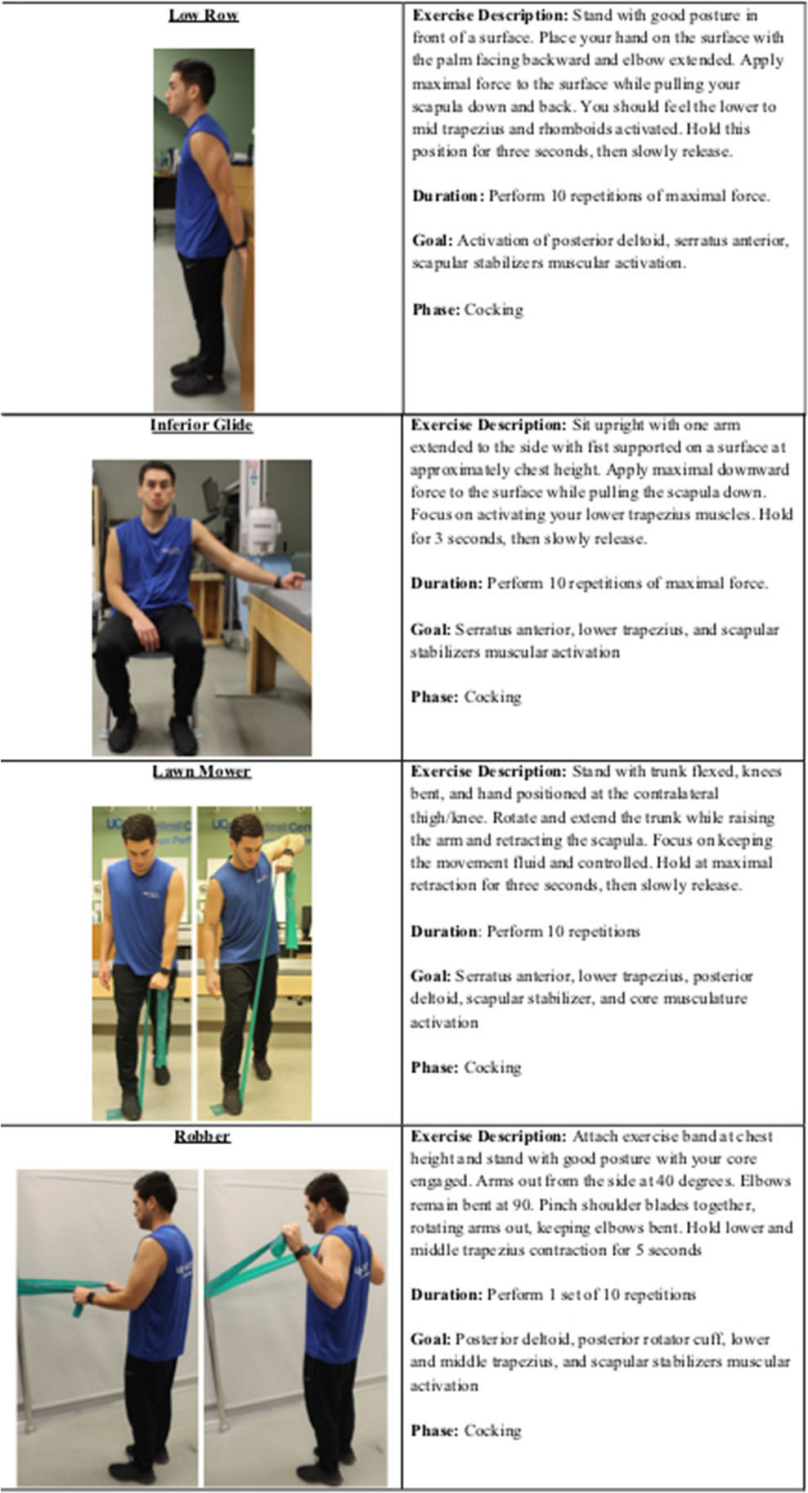
Incorporating these exercises into routine on-field training will promote appropriate scapular stabilization and glenoid positioning

Scapular stabilization exercises are effective both to treat and to prevent scapular dyskinesis and secondary shoulder injuries in throwing athletes. For athletes with scapular dyskinesis, exercises to counteract the abnormal protraction, depression, and rotation of the scapula are indicated [70, 71]. In healthy athletes, strengthening exercises for scapular stabilizers including the upper and lower trapezius and serratus anterior muscles promote normal scapular movement. The low row, inferior glide, lawnmower, and robbery exercises have been shown to effectively activate and strengthen these muscle groups in asymptomatic and symptomatic patients [68, 71, 72]. Incorporating these exercises into routine on-field training will promote appropriate scapular stabilization and glenoid positioning during the early cocking phase without disrupting the normal scapulohumeral rhythm (Figure 3).

## Late Cocking

During the late cocking phase, potential energy from the trunk is transferred to the throwing arm as it externally rotates and horizontally adducts. The late cocking phase prepares the arm for forward acceleration and subsequent ball release. The degree of external rotation achieved in this phase determines the range of forward movement in the following stages with greater external rotation leading to increased ball velocity [61]. The late cocking phase ends when the throwing arm is maximally externally rotated to 150 to 180° and horizontally adducted to approximately 20° [15, 62].

The infraspinatus and teres minor muscles contract concentrically to externally rotate the shoulder. As the arm externally rotates, the shoulder internal rotators (subscapularis, teres major, pectoralis major) contract eccentrically to control the speed of rotation. The posterior rotator cuff muscles (infraspinatus and teres minor) and latissimus dorsi contract to generate a posterior force which resists anterior humeral head translation and supports the anterior capsule [62]. The pectoralis major and anterior deltoid muscles also contract concentrically to horizontally adduct the throwing arm with peak angular velocity of 600° per second [15, 62].

Athletes often complain of shoulder pain in the late cocking position, where the throwing arm is abducted to 90°, the elbow is flexed to 90°, and the arm is maximally externally rotated in preparation for forward acceleration. In this position, the throwing arm holds its maximum potential energy [64]. Shoulder pain in this case can limit the athlete’s range of motion in external rotation and lead to decreased ball velocity during the acceleration phase [73]. Among professional pitchers, preseason deficits in external rotation are associated with in-season injury [74]. Pitchers with insufficient external rotation are more likely to be placed on the disabled list for a shoulder injury and require shoulder surgery than pitchers with deficits in internal or total shoulder rotation [75].

Having greater flexibility in external rotation can benefit throwing performance, but excessive stretching can exacerbate capsule laxity and lead to shoulder instability. Wilk refers to these competing interests as the “thrower’s paradox” [70, 76]. Athletes with inadequate external rotation may benefit from stretching exercises to promote increased range of motion, but repetitive throwing motions with extreme external rotation can lead to capsular instability and injury to the labrum or rotator cuff muscles [61]. For throwers with excessive laxity, one strategy to combat this paradox is increasing range of motion elsewhere in the chain. Increasing thoracic spine mobility in extension and rotation toward the pitching side may eliminate the need for excessive glenohumeral external rotation range of motion and help avoid resultant anterior capsule laxity and additional stress on the long head of the biceps tendon (Figure 3).

A proper warm-up routine to prevent pain with external rotation and capsular laxity during the late cocking phase should encompass thoracic spine mobility as well as facilitate activation of shoulder stabilizers including the middle and lower trapezius, rotator cuff musculature, biceps, and pec minor. With this in mind, we can address potential pain with external rotation and capsular laxity through the aforementioned thoracic spine rotation mobilization and thoracic spine extension mobilization in order to increase thoracic spine mobility toward the pitching side. In addition, the low row, inferior glide, lawn mower, and robber exercises can facilitate activation of the scapular stabilizing musculature [68, 71, 72].

## Acceleration

The acceleration phase takes place from maximal external rotation to the moment of ball release [47]. The potential energy accumulated by the throwing arm is utilized to accelerate the ball to its maximal velocity. The trunk flexes forward to neutral position as the throwing arm rotates internally and the elbow extends. Arm rotation lags behind elbow extension, with maximal elbow velocity occurring halfway through the acceleration phase and maximal internal rotation velocity occurring at ball release [3, 61, 77]. This delay reduces the inertia of the shoulder to increase the torque and angular velocity of the throwing arm [3]. The mean angular velocity of the arm at ball release is approximately 7,000° per second, making this one of the fastest human movements [3, 10, 47].

Muscle recruitment in this phase facilitates rapid rotation and extension of the arm. The quadriceps of the lead leg contracts concentrically to extend the leg. The trunk flexors (rectus abdominis, obliques) tilt the trunk forward to allow the throwing arm to accelerate through a greater distance [47, 61]. Internal rotation of the shoulder to 90 or 100° is achieved via concentric activation of the internal rotators (latissimus dorsi, pectoralis). The rotator cuff muscles, trapezius, serratus anterior, and levator scapulae remain active to stabilize the scapula and glenohumeral joint [15, 61]. Of note, rotator cuff and biceps activation is up to three times higher in amateur pitchers than in professional pitchers during this phase, potentially contributing to overuse injuries in young athletes [15]. Extension of the elbow during ball acceleration is achieved via the centrifugal force generated by the trunk and concentric activation of elbow extensors. Deceleration of elbow extension at ball release is achieved via eccentric activation of elbow flexors (biceps brachii, brachialis, and brachioradialis) [47, 61]. Wrist flexor muscles (flexor carpi radialis, flexor carpi ulnaris, and flexor digitorum) shift the wrist from hyperextension to neutral position at ball release [10, 47, 61].

Most elbow injuries among overhead athletes result from the substantial stresses applied to the elbow during the late cocking and acceleration phases. Injury to the ulnar collateral ligament (UCL) is common because the valgus stress applied to the medial elbow during ball acceleration exceeds the tensile strength of the UCL at 64 N⋅m [10, 78, 79]. Sidearm tracking due to decreased trunk flexion contributes to UCL trauma by increasing the force applied to the medial elbow [15]. UCL injury results in problems including elbow pain, decreased throwing velocity and control, joint instability, and muscle weakness [7, 10, 80]. Laxity of the UCL can also lead to additional ligamentous tears and ulnar nerve injury [79].

The medial sheer force of 300 N and compressive force of 500 N applied to the elbow can also result in valgus extension overload syndrome (VEO) with impingement of the posteromedial elbow [10, 79]. This syndrome is characterized by olecranon tip osteophytes, loose bodies, and damage to the posteromedial trochlea [79, 81]. Athletes with VEO often experience posterior elbow pain with elbow extension. VEO is common among baseball players who undergo elbow surgery with 65% of patients diagnosed with posterolateral olecranon osteophytes [82].

Elbow injury in overhand throwers often occurs secondary to adaptive changes in the shoulder which result in excessive force on the elbow. Pitchers often demonstrate an increase in external rotation, loss of internal rotation, and decrease in total range of motion of the throwing shoulder [83–87]. While these deficits are often described chronically, changes in shoulder range of motion have also been observed acutely after pitching. Reinold et al. described significant changes in shoulder and elbow range of motion within 30 min of pitching [85]. Kilber et al. similarly found significant and sustained loss of internal rotation up to 72 h after throwing [88]. Garrison and colleagues further demonstrated that deficits in total shoulder rotation range of motion are associated with UCL tears in high school and collegiate baseball players [84]. The acute time course of these adaptations suggests that stretching before and after a pitching session may be effective to prevent long-term changes.

**Figure 4:**
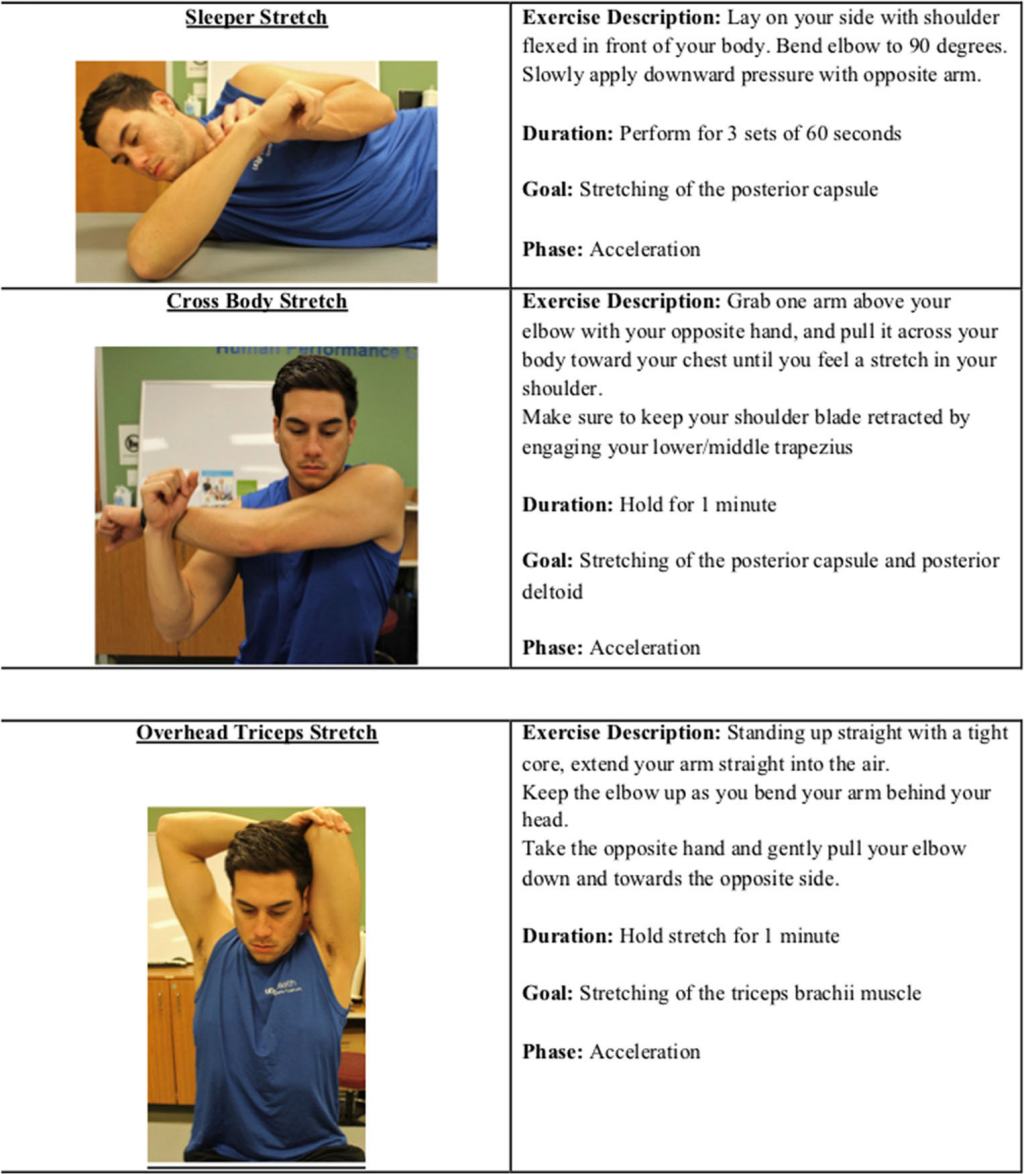
Stretches used to isolate the posterior aspect of the shoulder and triceps

Drills to prevent elbow injury in overhead athletes act by maintaining 180° total shoulder range of motion. The sleeper stretch is used to isolate the posterior aspect of the shoulder (posterior capsule, deltoid, and latissimus dorsi) and has been shown to effectively recover internal range of motion after pitching[89] (Figure 4). Other techniques including the cross-body stretch and overhead triceps stretch can also prevent glenohumeral internal rotation deficit (GIRD) [90] (Figure 4). A prevention program involving the cross-body stretch, overhead triceps stretch, and sleeper stretch among other exercises was found to significantly reduce the incidence of medial elbow injury and shoulder injury while improving hip and thoracic flexibility in youth pitchers [12, 91]. A separate study found that in a cohort of twenty pitchers, the two-out drill was effective to restore internal, external, and total range of motion of the throwing shoulder after a 40-pitch session, which can be incorporated in-game, between innings [92].

## Deceleration

The deceleration phase takes place between the time of ball release and maximal humeral head internal rotation with elbow extension [47]. During this last point of contact prior to completing the pitch, there are 0° of glenohumeral rotation, 100° of shoulder abduction, and 35° of horizontal adduction [93]. The teres minor, infraspinatus, and posterior deltoid are responsible for slowing the shoulder down and dissipating compressive forces across the joint [47]. Requiring a large eccentric contraction, the posterior musculature and posterior capsule are repeatedly placed in situations of potential injury [94]. In a clinical setting, these areas facing repeated trauma, coupled with continued anterior capsule stretching with the external rotation during the late cocking phase, pose a risk for glenohumeral internal rotation deficit (GIRD).

The biceps brachii and brachialis are also active during deceleration by contracting eccentrically to slow down elbow extension and forearm pronation [3]. Other structures to take note of are the trapezius, rhomboids, and serratus anterior, as they all assist in the deceleration phase and help the thrower stabilize their scapula throughout movement [47]. Without proper kinematics, muscle activation, and form, there will be a higher risk of injury during this time. A comprehensive warm-up routine is one avenue to combat this increased injury risk.

A focus on scapular stability and posterior deltoid strength is paramount for a successful deceleration phase in order to combat the extreme rotational and distraction forces being placed on the shoulder. GIRD, along with subacromial impingement, biceps brachii tendinitis, posterior rotator cuff tendinitis, and posterior capsule tightening are just some of the injuries that can be seen within a baseball throwing shoulder as a result of overuse, and stress placed during this phase.

**Figure 5:**
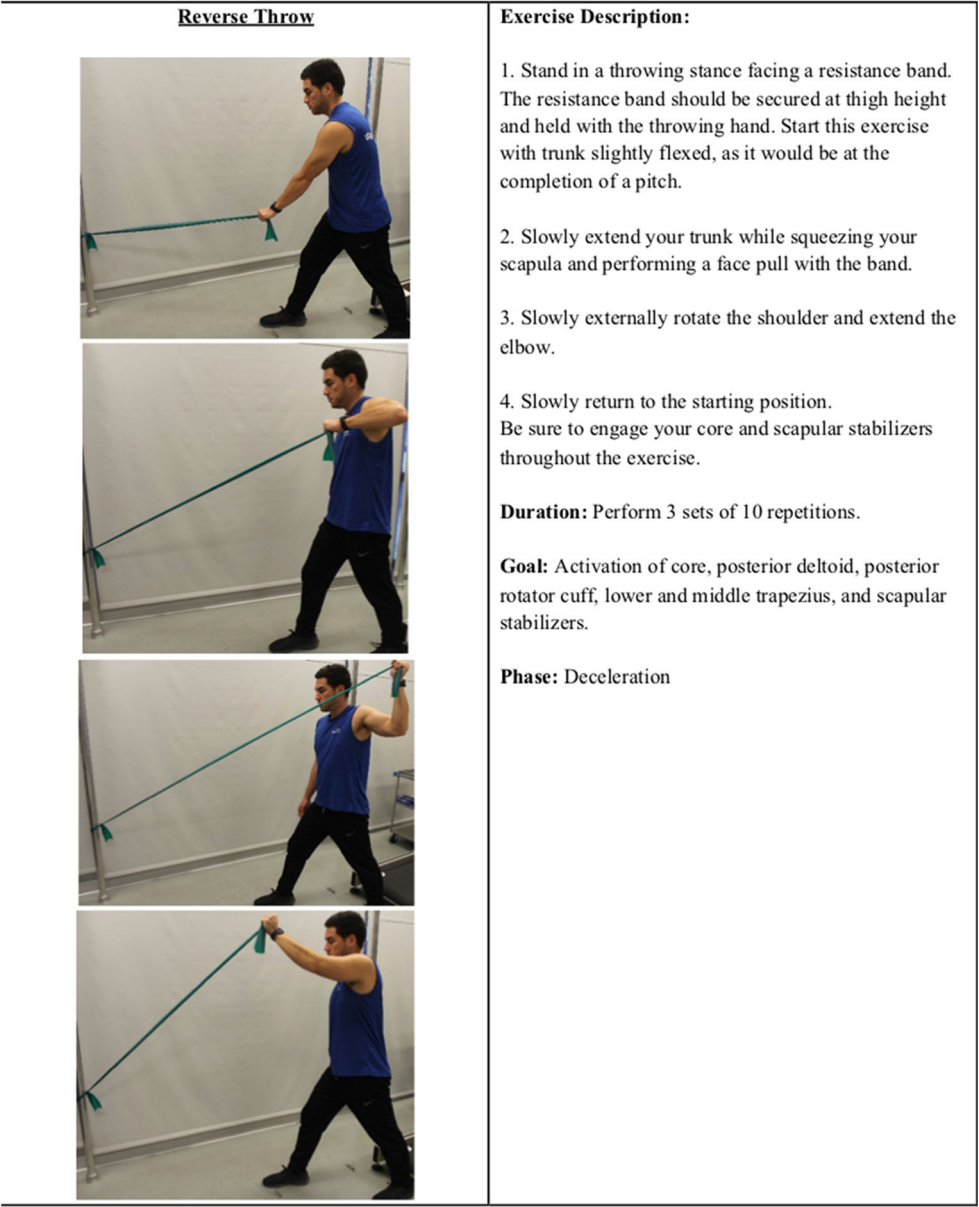
A banded reverse throw allows for activation of the scapular stabilizers, warming up the posterior deltoid and rotator cuff, and preparing the shoulder to decelerate a throw by eccentrically resisting a band

The low row, inferior glide, lawnmower, and robbery exercises as used in the early cocking phases are helping prime the shoulder for the violent motion of a pitch and activate the musculature stated above. Additionally, a banded reverse throw allows for activation of the scapular stabilizers, warming up the posterior deltoid and rotator cuff, and preparing the shoulder to decelerate a throw by eccentrically resisting a band (Figure 5).

## Follow-through

The follow-through phase is the culmination of the kinetic chain: linking together, generating forces, and delivering the pitch. Due to decreased joint loading during this phase, the risk of injury is reduced relative to other phases. The pitcher will simply continue to move forward toward the catcher until arm motion has ceased, and then become a fielder for the remainder of the play.

## Conclusion

The throwing cycle is a complex motion that places various stresses throughout the thrower’s body, from the ankle to the core, and from the back to the shoulder and elbow. A thorough understanding of the mechanics of this motion, along with specific exercises to target the specific actions of each phase as described here, may allow for throwers to minimize injury risk (Table 1).

**Table 1:** A thorough understanding of the mechanics of this motion, along with specific exercises to target the specific actions of each phase as described here, may allow for throwers to minimize injury risk

Phase	Notable active musculature	Potential concerns	Warm-up exercises
Windup	Iliopsoas, rectus femoris, pectineus, sartorius, tensor fascia latae, gluteus medius, gluteus minimus, core	Center of gravity, ankle dorsiflexion, lumbopelvic control	Ankle box stretch, lateral monster walk, forward lunge, lateral lunge, bird dog
Stride	Tensor fascia latae, gluteus medius, gluteus minumus, core	Lumbar hypermobility, Thoracic hypomobility	Pallof press, lift, chop, thoracic spine extension mobilization, thoracic spine rotation mobilization
Cocking	Deltoid, rotator cuff, levator scapulae, serratus anterior, trapezius, rhomboid, pectoralis minor	Scapular dyskinesis, glenohumeral capsular laxity	Low row, inferior glide, lawn mower, robber
Acceleration	Latissimus dorsi, pectoralis, rotator cuff, trapezius, serratus anterior, levator scapulae, biceps brachii	Glenohumeral internal rotation deficit	Sleeper stretch, cross body stretch, overhead triceps stretch
Deceleration	Teres minor, infraspinatus, posterior deltoid, biceps brachii, brachialis, trapezius, rhomboid, serratus anterior	Glenohumeral internal rotation deficit, subacromial impingement, biceps brachii tendinitis, posterior rotator cuff tendinitis, posterior capsule tightening	Reverse throw
Follow-through	Culmination of kinetic chain delivering the pitch. Low risk of injury during this phase		
